# Structural characterization and phylogenetic analysis of the chloroplast genome of *Monarda didyma* (Lamiaceae)

**DOI:** 10.1080/23802359.2026.2758482

**Published:** 2026-11-03

**Authors:** Chunquan Cao, Guoyu Gao, Hongqin Li, Xiujuan Yin, Jiatong Jin, Liqiang Wang

**Affiliations:** College of Pharmacy, Heze University, Heze, Shandong Province, P. R. China

**Keywords:** Chloroplast genome, Lamiaceae, *Monarda didyma*, phylogenetic evolution

## Abstract

*Monarda didyma* is an aromatic herb native to eastern North America with ecological, medicinal, and ornamental value, but its chloroplast genome has not been characterized. Here, we assembled its complete chloroplast genome (151,812 bp) using next-generation data. The genome exhibits a typical quadripartite structure (LSC: 83,025 bp; SSC: 17,663 bp; IRs: 25,562 bp each), contains 132 genes (87 protein-coding, 37 tRNA, 8 rRNA genes) and 37.82% GC content. Phylogenomic analysis resolved *M. didyma* and *Pycnanthemum tenuifolium* as a fully supported sister clade (BS = 100%). This genome provides a resource for species identification and phylogenetic studies of *Monarda* and related genera.

## Introduction

*Monarda didyma* Linnaeus (1753), commonly known as bee balm or Oswego tea, is an herbaceous perennial in the Lamiaceae, native to eastern North America (POWO 2026; USDA and NRCS 2009). The species is valued for its essential oil, rich in aromatic and phenolic monoterpenes with documented antioxidant, anti-inflammatory and antimicrobial activities (Fraternale et al. 2022; Gontar et al. 2025; Cichosz et al. 2026). Different organs and developmental stages significantly influence the chemical composition of its essential oil and hydrosol (Gontar et al. 2025).

Chloroplast genomes are accumulating rapidly across Lamiaceae. Recent studies characterized three Iranian *Teucrium* species and identified *rpo*C2, *ycf*1, *ndh*F, *rpl*32, *rps*16, *acc*D and *trn*H-GUG–*psb*A spacer as candidate markers (Hejazi et al. 2025); reported the first plastome of Iranian *Mentha aquatica*, with *ycf*1 and *rpl*2–*trn*H as the most polymorphic regions (Soorni and Golchini 2025); and analyzed five Iranian *Salvia* plastomes, validating *rpl*14–*rpl*16 and *psb*K–*psb*I as effective markers (Akrami et al. 2025). These studies confirm the power of plastome in Lamiaceae systematics, but sampling remains biased toward *Salvia*, *Mentha* and *Teucrium*.

For *Monarda*, genomic resources remain limited. NCBI currently holds only a few gene sequences, such as ITS (e.g. MN636287), *mat*K (e.g. MG224897) and *rbc*L (e.g. MN642630), and no complete chloroplast genome has been reported. Consequently, the phylogenetic placement of *Monarda* relative to *Pycnanthemum* and *Clinopodium* has not been tested with genome-scale chloroplast data, and the generic delimitation of *Clinopodium* sensu lato—shown to be non-monophyletic (paraphyletic) in molecular phylogenetic analyses (Bräuchler et al. 2010; Drew and Sytsma 2012), with nuclear–plastid conflict leaving its circumscription unresolved (Bräuchler et al. 2010)—also remains untested at this scale.

Herein, we assemble and annotate the first complete chloroplast genome of *M. didyma*, compare its structure with other Lamiaceae chloroplast genomes, and reconstruct its phylogenetic position, providing a genomic resource for *Monarda* and related genera.

## Materials and methods

### Plant materials and DNA extraction

Healthy fresh leaves were obtained from a cultivated population in Peony District, Heze City, Shandong Province, China (115.45615°E, 35.27039°N) (Figure 1). The plant was identified by Liqiang Wang based on morphological characters. The source is a locally maintained garden population; no records document its introduction to Heze. Voucher (HZU-2025-0804) is deposited at the Herbarium of Heze University.

**Figure 1.**
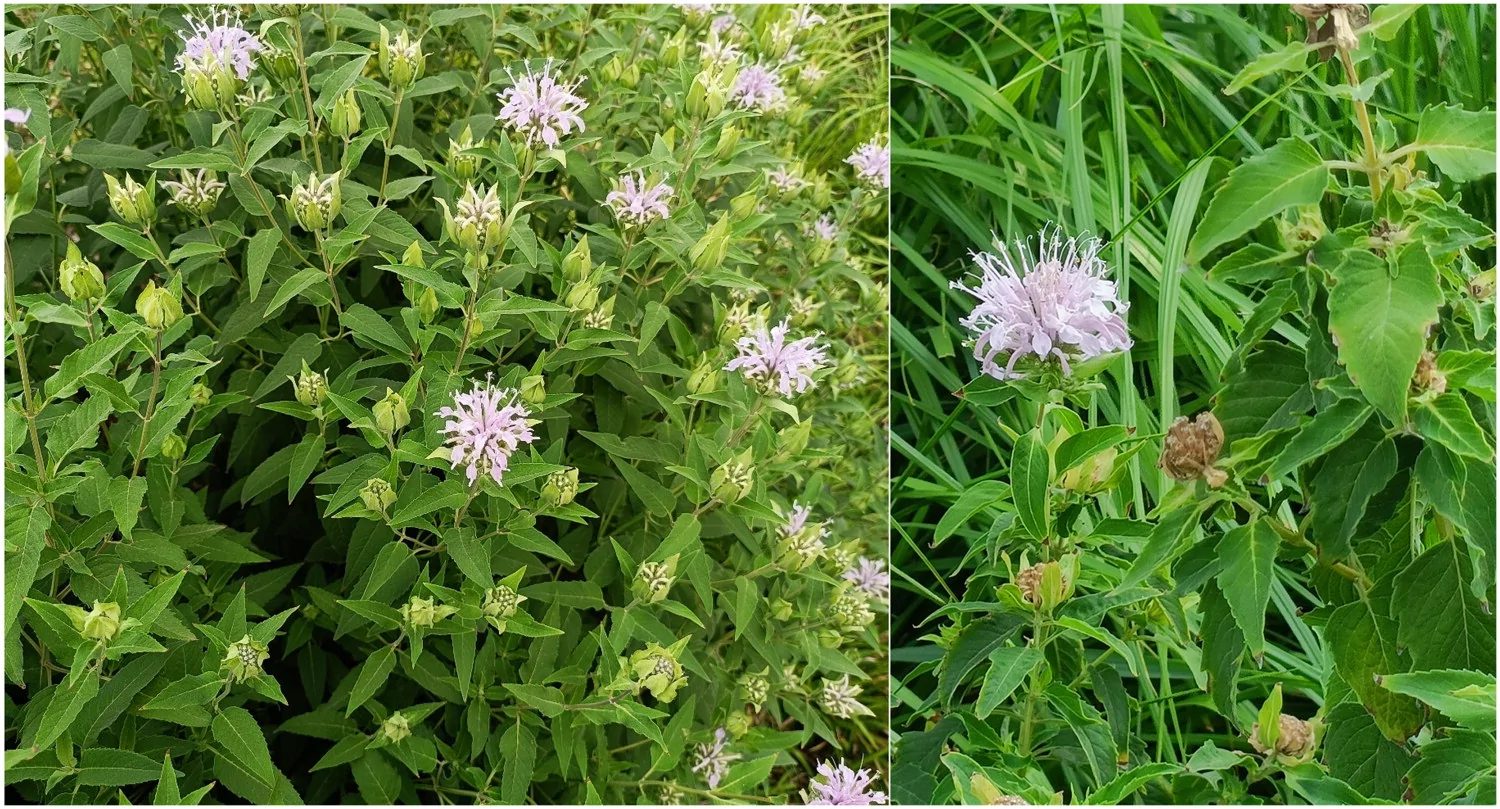
Field photograph of *Monarda didyma*. A cultivated plant from Peony District, Heze City, Shandong, China, showing a pale lavender-pink corolla. *Monarda didyma* is an erect herbaceous perennial, 70–150 cm tall, with sharply quadrangular stems, opposite ovate to ovate-lanceolate leaves, and dense terminal capitate inflorescences. Flower color ranges from red to pink, purple and white (POWO 2026; Royal Horticultural Society n.d.), and pink-flowered cultivars are common in horticulture. Identified and photographed by Liqiang Wang.

### Genome sequencing, assembly and annotation

Whole genomic DNA was extracted using the Plant Genomic DNA Kit (Tiangen Biotech, Beijing, China), sheared into 200–400 bp fragments for library construction, and sequenced on the DNBSEQ platform (150 bp paired-end) (Benagen Tech, Wuhan, China). Raw reads were filtered with Trimmomatic (v0.35) (Bolger et al. 2014), and ∼28 Gb of clean reads were assembled with GetOrganelle (v1.7.1) (Jin et al. 2020) using ‘-k 21,45,65,85,105 -R 100 -F embplant_pt’. The complete chloroplast genome was annotated with CPGAVAS2 (Shi et al. 2019), manually refined in Apollo (Lewis et al. 2002), and visualized the circular genome and cis/trans- splicing gene structure using CPGview (Liu et al. 2023). Sequencing depth and coverage map was calculated with DrawSeqDepth (https://github.com/wlqg1983/DrawSeqDepth) using default parameters.

### Phylogenetic analysis

To investigate the phylogenetic position of *M. didyma* within Mentheae, chloroplast genomes of 24 other Lamiaceae species were retrieved from GenBank. *Ajuga forrestii* (MN518848) (Hao et al. 2023) and *Ajuga bracteosa* (OM630151) (Seo and Kim 2026) were used as outgroups, as both belong to Ajugoideae, which lies outside the Nepetoideae containing Mentheae (Zhao et al. 2021). Seventy-two shared protein-coding genes (PCGs) were extracted, aligned with MAFFT (Katoh and Standley 2016), and concatenated. A maximum-likelihood (ML) tree was constructed using IQ-TREE (v2.0) (Nguyen et al. 2015) under TVM+F + I + R5 with 1000 bootstrap replicates.

### RSCU and Pi analysis

Relative Synonymous Codon Usage (RSCU) was calculated in PhyloSuite (v2) (Zhao et al. 2025) and visualized *via* the RSCU-Plot Shiny app (https://pcg-lab.shinyapps.io/RSCU-Plot/) (Diani Gohar and Soorni 2025). Nucleotide diversity (Pi) was estimated using CPStools (Huang et al. 2024).

## Results

The chloroplast genome of *M. didyma* is 151,812 bp in length and displays a typical quadripartite structure (Figure 2). The assembled genome achieved an average sequencing depth of 4,335.10×, with a range of 1,691× to 6,296× (Figure S1). The chloroplast genome exhibits a variable GC content, with an overall value of 37.82%, ranging from 31.73% in the SSC region to 43.05% in the IR regions, and 35.9% in the LSC region. A total of 132 genes were predicted, including 87 PCGs, eight rRNA genes, and 37 tRNA genes. Seven unique PCGs (*rpl*2, *rpl*23, *ycf*2, ycf15, *ndh*B, *rps*7, and *rps*12), six unique tRNA genes (*trn*I-CAU, *trn*L-CAA, *trn*V-GAC, *trn*A-UGC, *trn*R-ACG, and *trn*N-GUU), and four unique rRNA genes (*rrn*16S, *rrn*23S, *rrn*4.5S, and *rrn*5S) were located in IR regions. Within the entire genome, nine PCGs (*rps*16, *atp*F, *rpoC*1, *pet*B, *pet*D, *rpl*16, *rpl*2, *ndh*A and *ndh*B) each contained one intron, while three PCGs (*clp*P, *rps*12 and *ycf*3) contained two introns. Additionally, six tRNA genes (*trn*K-UUU, *trn*G-UCC, *trn*L-UAA, *trn*V-UAC, *trn*I-GAU and *trn*A-UGC) contained one intron. The *rps*12 gene was identified as a trans-splicing gene. The structures of cis-/trans- splicing PCGs were presented in Figures S2 and S3.

**Figure 2.**
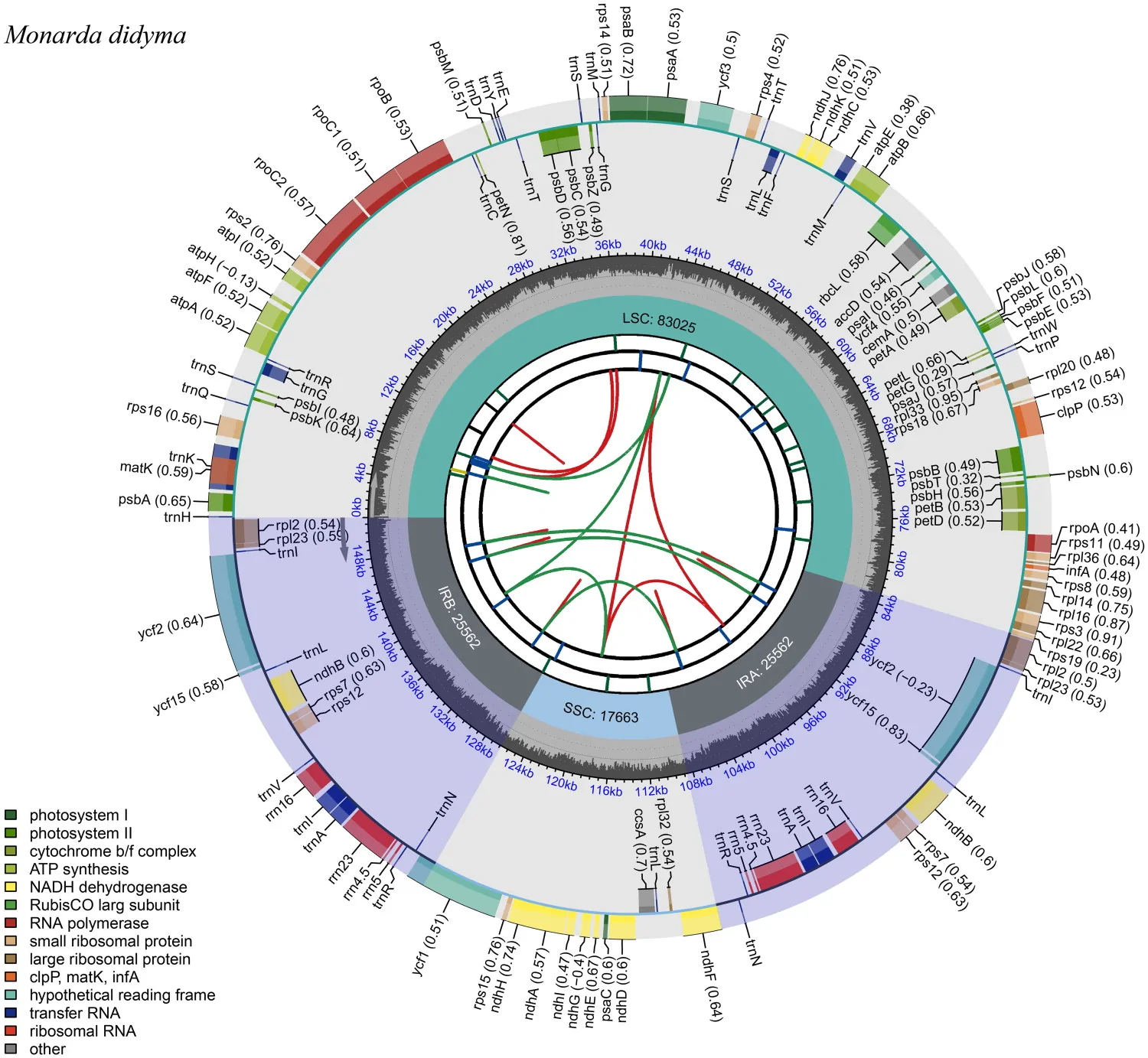
The complete chloroplast genome map of *Monarda didyma*. The map contains six tracks. From the center outward, the first track shows the dispersed repeats. The second track displays long tandem repeats as short blue bars. The third track illustrates SSRs as short bars with various colors. The small single-copy (SSC), inverted repeat (IRA and IRB), and large single-copy (LSC) regions are represented in the fourth track. The GC content along the genome is visualized in the fifth track. The genes are shown in the sixth track, with genes from different functional groups color-coded. Genes transcribed in a clockwise direction are located on the outside of the map, while genes transcribed counterclockwise are shown on the inside.

**Figure 3.**
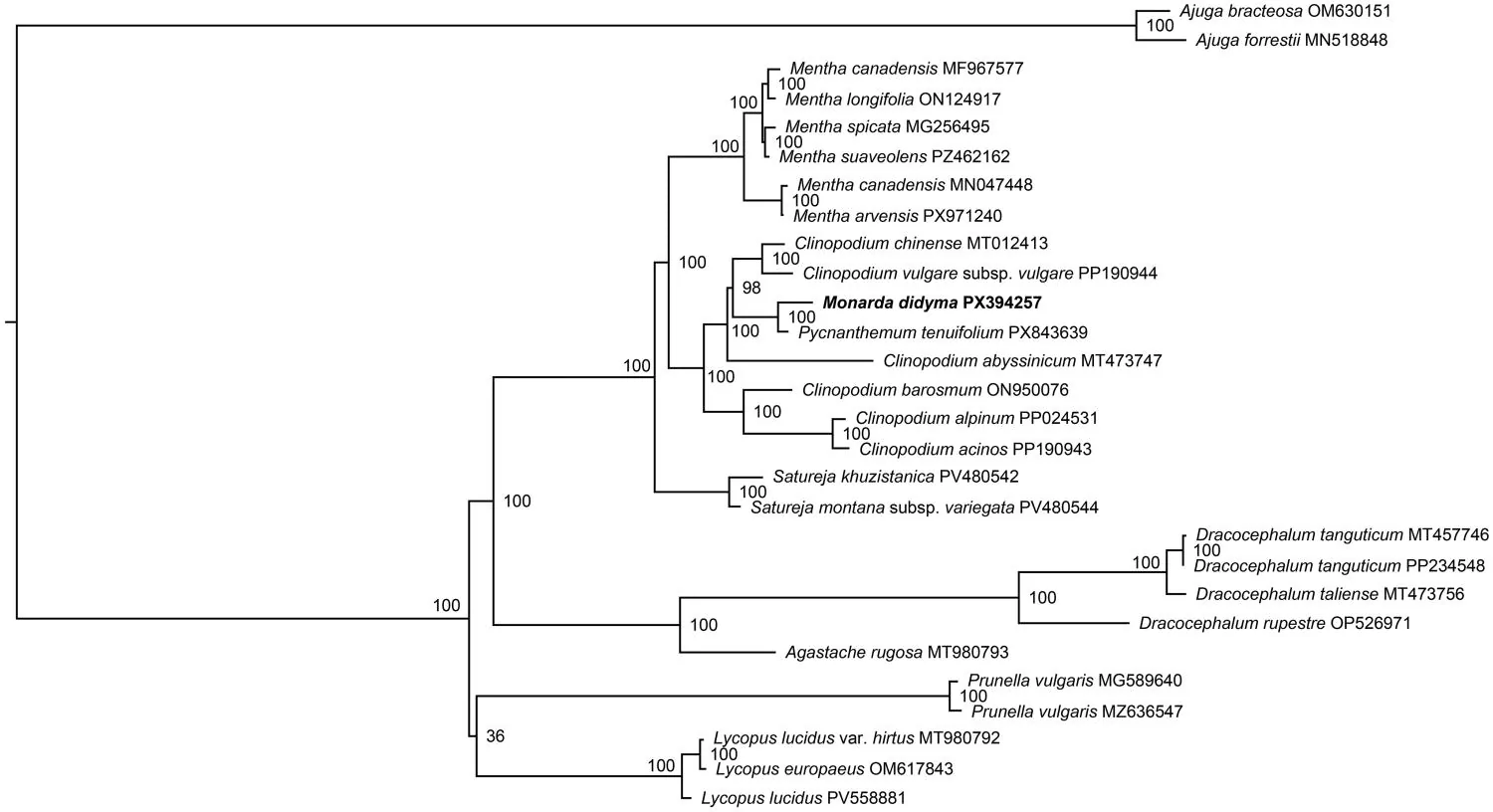
Maximum-likelihood phylogeny of *Monarda didyma* and its close relatives. The bootstrap values from 1000 replicates were shown for each node in the phylogenetic tree. The 26 species were downloaded from the GenBank: *Ajuga forrestii* MN518848 (outgroup) (Tao et al. 2019), *Ajuga bracteosa* OM630151 (outgroup) (Shang et al. 2023), *Lycopus lucidus* PV558881, *Lycopus lucidus* var. *hirtus* MT980792 (Wang et al. 2021), *Lycopus europaeus* OM617843 (Du et al. 2022), *Prunella vulgaris* MZ636547, *Prunella vulgaris* MG589640 (Han and Zheng 2018), *Agastache rugosa* MT980793 (Wang et al. 2021), *Dracocephalum rupestre* OP526971 (Han et al. 2023), *Dracocephalum tanguticum* PP234548, *Dracocephalum taliense* MT473756 (Zhao et al. 2021), *Satureja montana* subsp.* variegata* PV480544 (Diani Gohar and Soorni 2025), *Satureja khuzistanica* PV480542 (Diani Gohar and Soorni 2025), *Mentha canadensis* MN047448 (Li et al. 2020), *Mentha arvensis* PX971240, *Mentha suaveolens* PZ462162, *Mentha spicata* MG256495 (Wang et al. 2017), *Mentha longifolia* ON124917 (Zubair Filimban et al. 2022), *Mentha canadensis* MF967577, *Clinopodium barosmum* ON950076, *Clinopodium alpinum* PP024531, *Clinopodium acinos* PP190943, *Clinopodium abyssinicum* MT473747 (Zhao et al. 2021), *Pycnanthemum tenuifolium* PX843639, *M. didyma* PX394257 (the new reported chloroplast genome labeled by bold font), *Clinopodium vulgare* subsp. *vulgare* PP190944, *Clinopodium chinense* MT012413 (Zhang et al. 2020).

Phylogenetic reconstruction based on complete chloroplast genomes placed *M. didyma* within a well-resolved topology (Figure 3). *Monarda didyma* and *Pycnanthemum tenuifolium* formed a fully supported sister clade (BS = 100%), nested within *Clinopodium*, and all *Clinopodium* species together with the clade formed a monophyletic group. Based on the clade and *Clinopodium* species, Pi analysis identified *trn*T-GGU (π = 0.10075), *ycf*1 (π = 0.01828), and *ndh*F (0.01367) as the most variable genes, and *psb*I*–trn*S-GCU (0.05183), *trn*H-GUG*–psb*A (0.04467) and *ccs*A*–ndh*D (0.04625) as the most variable intergenic regions (Figure S4), suggesting their potential as DNA barcodes. RSCU analysis showed a strong preference for A/U-ending codons (e.g. Leu UUA ≥ 1.85; Arg AGA ≥ 1.82), whereas G/C-ending codons were less frequent (Figure S5).

## Discussion and conclusion

We report the first complete chloroplast genome of *M. didyma*, a species in the subfamily Nepetoideae, tribe Mentheae (Lamiaceae). With a length of 151,812 bp and 132 predicted genes, this genome exhibits the typical quadripartite structure commonly found in land plant chloroplast genomes.

Our phylogenetic analyses revealed that *M. didyma* and *Pycnanthemum tenuifolium* formed a strongly supported sister clade (BS = 100%), which is consistent with the ITS-based study of Prather et al. (2002), further confirming the close relationship between *Monarda* and *Pycnanthemum*. Notably, this clade did not emerge as an independent lineage but was instead nested within *Clinopodium* (closely related to *C. chinense* and *C. vulgare* subsp. *vulgare*). This placement suggests that *Clinopodium* sensu lato is paraphyletic if *Monarda* and *Pycnanthemum* are maintained as distinct genera, adding to the growing evidence that its generic boundaries remain problematic (Bräuchler et al. 2010; Janković et al. 2025).

Using chloroplast (*trn*K intron, *trn*L*-*F) and nuclear (ITS) sequence data, Bräuchler et al. (2010) showed that *Clinopodium* s.l. is a highly unnatural assemblage, with species from more than 20 genera intermingled within it. Drew and Sytsma (2012) also recovered *Clinopodium* as paraphyletic within tribe Mentheae. Our study, based on complete chloroplast genome data, provides independent genome-scale evidence that reinforces these conclusions. Given the non-monophyly of *Clinopodium* as currently circumscribed and the nesting of *Monarda* and *Pycnanthemum* within it, future studies that expand taxon sampling of *Clinopodium* and integrate morphological and multi-locus molecular data are needed to comprehensively reassess the generic delimitation of this taxonomically complex group.

## Supplementary Material

Supplemental Material

Supplemental Material

Supplemental Material

Supplemental Material

Supplemental Material

## Data Availability

The complete chloroplast genome sequence of *M. didyma* in this study has been submitted to the NCBI database (https://www.ncbi.nlm.nih.gov) under the accession number PX394257.1. The associated BioProject, BioSample, and SRA numbers are PRJNA1146888, SAMN62196194, and SRR40014555.
